# {6,6′-Dimeth­oxy-2,2′-[*o*-phenyl­enebis(nitrilo­methyl­idyne)]diphenolato}cobalt(II) dichloro­methane disolvate

**DOI:** 10.1107/S160053680900083X

**Published:** 2009-01-14

**Authors:** Atsuhiro Nabei, Takayoshi Kuroda-Sowa, Takashi Okubo, Masahiko Maekawa, Megumu Munakata

**Affiliations:** a Department of Chemistry, Kinki University, 3-4-1, Kowakae, Higashi-Osaka, Osaka 577-8502, Japan

## Abstract

The title compound, [Co(C_22_H_18_N_2_O_4_)]·2CH_2_Cl_2_, was isolated from the reaction of *N*,*N*′(*o*-phenyl­ene)bis­(vanillalimine) (H_2_ *L*) with Co(SCN)_2_. The crystal structure contains a Co^II^ ion surrounded by the *L* ^2−^ ligand in a slightly distorted square-planar fashion. Inter­molecular C—H⋯O hydrogen-bonding contacts between the dichloro­methane solvent mol­ecules and the meth­oxy or carboxyl­ate O atoms are observed in the crystal structure. The planar complex mol­ecules stack through inversion related π–π inter­actions between the six-membered rings of the vanillalimine half ligands. The distance between centroids is 3.498 (2) Å and the perpendicular distance is 3.345 Å. A partial stacking is observed with a centroid–centroid distance of 3.830 (2) Å, a perpendicular distance of 3.350 Å and a slippage of 1.856 Å.

## Related literature

For general background, see: Cotton *et al.* (1999 ▶); Liu *et al.* (2007 ▶); Sharghi & Al Nasseri (2003 ▶). For related structures, see: Pahor *et al.* (1976 ▶). For related properties, see: Bella *et al.* (1995 ▶).
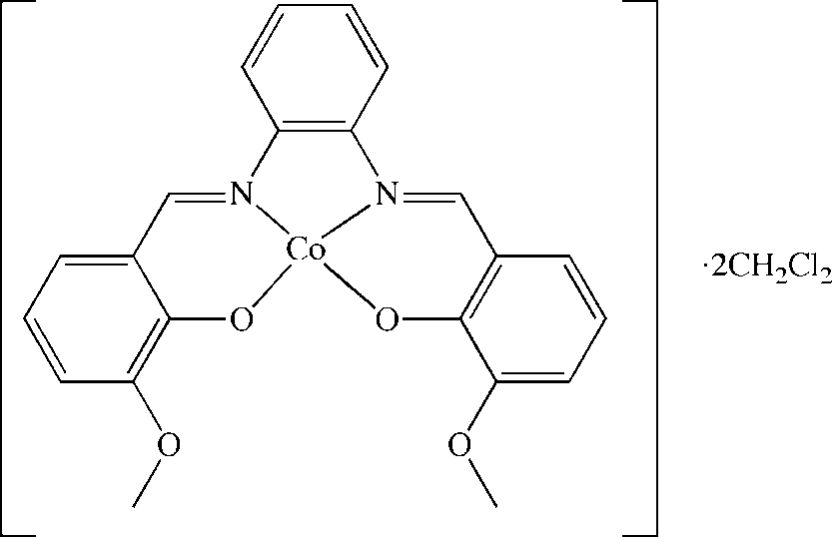


## Experimental

### 

#### Crystal data

[Co(C_22_H_18_N_2_O_4_)]·2CH_2_Cl_2_
*M* *_r_* = 603.19
Monoclinic, 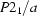

*a* = 13.309 (6) Å
*b* = 14.088 (6) Å
*c* = 14.101 (6) Å
β = 109.676 (6)°
*V* = 2489.4 (19) Å^3^
*Z* = 4
Mo *K*α radiation
μ = 1.15 mm^−1^
*T* = 150 (1) K
0.50 × 0.30 × 0.10 mm

#### Data collection

Rigaku Mercury diffractometer
Absorption correction: multi-scan (*REQAB*; Jacobson, 1998 ▶) *T*_min_ = 0.764, *T*_max_ = 0.891
28457 measured reflections
5677 independent reflections
5100 reflections with *F* ^2^ > 2σ(*F* ^2^)
*R*_int_ = 0.040

#### Refinement

*R*[*F* ^2^ > 2σ(*F* ^2^)] = 0.046
*wR*(*F* ^2^) = 0.089
*S* = 1.15
5677 reflections
317 parameters
H-atom parameters constrained
Δρ_max_ = 0.53 e Å^−3^
Δρ_min_ = −0.66 e Å^−3^

### 

Data collection: *CrystalClear* (Rigaku/MSC, 2007 ▶); cell refinement: *CrystalClear*; data reduction: *CrystalStructure* (Rigaku/MSC, 2007 ▶); program(s) used to solve structure: *SIR97* (Altomare *et al.*, 1999 ▶); program(s) used to refine structure: *SHELXL97* (Sheldrick, 2008 ▶); molecular graphics: *ORTEPII* (Johnson, 1976 ▶); software used to prepare material for publication: *CrystalStructure*.

## Supplementary Material

Crystal structure: contains datablocks global, I. DOI: 10.1107/S160053680900083X/si2147sup1.cif

Structure factors: contains datablocks I. DOI: 10.1107/S160053680900083X/si2147Isup2.hkl

Additional supplementary materials:  crystallographic information; 3D view; checkCIF report

## Figures and Tables

**Table 1:** Selected bond lengths (Å)

Co1—O1	1.8593 (15)
Co1—O2	1.8611 (14)
Co1—N1	1.8807 (17)
Co1—N2	1.8755 (18)

**Table 2:** Hydrogen-bond geometry (Å, °)

*D*—H⋯*A*	*D*—H	H⋯*A*	*D*⋯*A*	*D*—H⋯*A*
C23—H23*A*⋯O3	0.99	2.54	3.472 (4)	158
C23—H23*B*⋯O4	0.99	2.33	3.252 (4)	154
C23—H23*B*⋯O2	0.99	2.41	3.202 (3)	137
C24—H24*A*⋯O3	0.99	2.35	3.173 (4)	140
C24—H24*B*⋯O4	0.99	2.38	3.250 (4)	147

**Fig. 1.**
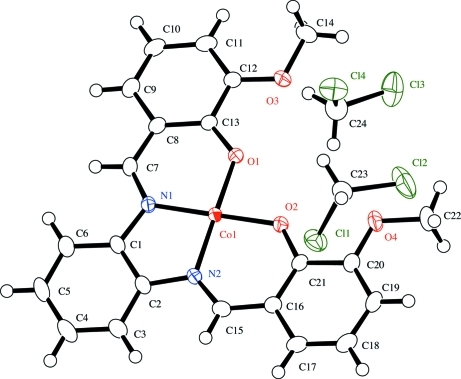
The molecular structure of (I), with the atom-numbering scheme, showing 50% probability displacement ellipsoids.

## supplementary crystallographic information

### Comment

Cobalt–Schiff base complexes are thoroughly investigated in their catalytic oxidation, chiral synthesis in solution, and so on (Cotton *et al.*, 1999; Liu *et al.*, 2007; Sharghi *et al.*, 2003). However, a relatively few works are devoted to their solid state properties such as magnetic or conductive properties (Bella *et al.*, 1995). Our interest on the cobalt-salophen system is based on their planar structure (Pahor *et al.*, 1976) together with its redox-active characteristics, which implies their potential application for conducting and/or magnetic devices. In this work the structure of the title molecule, [Co(C_22_H_18_N_2_O_4_)]^.^2(CH_2_Cl_2_) is reported (Fig. 1) The coordination sphere around Co^II^ ions is square planar and made up of equatorial planar N_2_O_2_ coordination from *L*^2-^. The Co—O and Co—N bond distances are given in Table 1. The Co—N distances are comparable to those of low spin (LS) Co^II^ species, representing a characteristic feature of the LS state. Intermolecular C—H···O hydrogen bonding contacts are observed in the title structure (Table 2) between methoxy oxygen atoms and the dichloromethane donors. The planar molecules stack each other through inversion related π···π interactions, with the centroids *Cg*3···*Cg*6^i^ distance of 3.498 (2) Å, the perpendicular distance of the centroid *Cg*3 and the plane of *Cg*6 is 3.345 Å; a partial stacking is also observed between inversion related *Cg*3 rings: *Cg*3···*Cg*3^i^ = 3.830 (2) Å, the perpendicular distance is 3.350 Å, the slippage between rings is 1.856 Å, symmetry code i = -*x*, -*y*, 1 - *z*. definitions: *Cg*3 is the centroid of the ring Co, O2, C21, C16, C15, N2 *Cg*6 is the centroid of the ring C16 - C21

### Experimental

To a dichloromethane solution (10 ml) containing H_2_L (0.1 mmol, 42.7 mg) placed at the bottom of a glass tube, a methanol solution (10 ml) containing Co(SCN)_2_ (0.1 mmol, 17.6 mg) was added quietly. After standing for two weeks at room temperature, brown brick crystals of (I) suitable for X-ray analysis were obtained.

### Refinement

All H atoms were positioned geometrically and treated as riding [C—H = 0.95 Å, 0.98 Å and 0.99 Å, and *U*_iso_(H)= 1.2*U*_eq_(C)].

### Crystal data

**Table:**

[Co(C_22_H_18_N_2_O_4_)]·2CH_2_Cl_2_	*F*(000) = 1228.00
*M**_r_* = 603.19	*D*_x_ = 1.609 Mg m^−^^3^
Monoclinic, *P*2_1_/*a*	Mo *K*α radiation, λ = 0.71070 Å
Hall symbol: -P 2yab	Cell parameters from 6959 reflections
*a* = 13.309 (6) Å	θ = 3.1–27.5°
*b* = 14.088 (6) Å	µ = 1.15 mm^−^^1^
*c* = 14.101 (6) Å	*T* = 150 K
β = 109.676 (6)°	Block, brown
*V* = 2489.4 (19) Å^3^	0.50 × 0.30 × 0.10 mm
*Z* = 4	

### Data collection

**Table:**

Rigaku Mercury diffractometer	5100 reflections with *F*^2^ > 2σ(*F*^2^)
Detector resolution: 7.31 pixels mm^-1^	*R*_int_ = 0.040
ω scans	θ_max_ = 27.5°
Absorption correction: multi-scan (*REQAB*; Jacobson, 1998)	*h* = −17→17
*T*_min_ = 0.764, *T*_max_ = 0.891	*k* = −18→17
28457 measured reflections	*l* = −18→18
5677 independent reflections	

### Refinement

**Table:**

Refinement on *F*^2^	H-atom parameters constrained
*R*[*F*^2^ > 2σ(*F*^2^)] = 0.046	*w* = 1/[σ^2^(*F*_o_^2^) + (0.0308*P*)^2^ + 1.9552*P*] where *P* = (*F*_o_^2^ + 2*F*_c_^2^)/3
*wR*(*F*^2^) = 0.089	(Δ/σ)_max_ < 0.001
*S* = 1.15	Δρ_max_ = 0.53 e Å^−^^3^
5677 reflections	Δρ_min_ = −0.66 e Å^−^^3^
317 parameters	

### Special details

**Table:**

Geometry. The planar Co^II^ complex has non-crystallographic mm2 symmetry.
Refinement. Refinement was performed using all reflections. The weighted *R*-factor (*wR*) and goodness of fit (*S*) are based on *F*^2^. *R*-factor (gt) are based on *F*. The threshold expression of *F*^2^ > 2.0 σ(*F*^2^) is used only for calculating *R*-factor (gt).

### Fractional atomic coordinates and isotropic or equivalent isotropic displacement parameters (Å^2^)

**Table:**

	*x*	*y*	*z*	*U*_iso_*/*U*_eq_	
Co1	0.13131 (2)	0.19517 (2)	0.52772 (2)	0.01696 (7)	
Cl1	0.44427 (5)	0.14026 (5)	0.68960 (5)	0.03951 (16)	
Cl2	0.47220 (7)	0.09420 (8)	0.89664 (6)	0.0672 (2)	
Cl3	0.21820 (7)	0.13146 (7)	0.96127 (7)	0.0621 (2)	
Cl4	−0.00594 (6)	0.12289 (5)	0.83886 (6)	0.04414 (17)	
O1	0.17603 (12)	0.26802 (11)	0.64445 (11)	0.0208 (3)	
O2	0.16176 (12)	0.08953 (10)	0.61189 (11)	0.0208 (3)	
O3	0.24984 (13)	0.33830 (12)	0.82700 (11)	0.0269 (3)	
O4	0.21621 (14)	−0.03363 (12)	0.75913 (12)	0.0310 (3)	
N1	0.09468 (14)	0.30090 (13)	0.44144 (13)	0.0186 (3)	
N2	0.09571 (14)	0.12300 (13)	0.40921 (13)	0.0186 (3)	
C1	0.05949 (17)	0.27635 (16)	0.33749 (16)	0.0202 (4)	
C2	0.06502 (17)	0.17945 (16)	0.32009 (16)	0.0207 (4)	
C3	0.04319 (19)	0.14651 (18)	0.22208 (17)	0.0277 (5)	
C4	0.0107 (2)	0.2098 (2)	0.14228 (18)	0.0329 (5)	
C5	−0.00149 (19)	0.3053 (2)	0.15966 (18)	0.0315 (5)	
C6	0.02351 (19)	0.33956 (18)	0.25698 (18)	0.0273 (5)	
C7	0.10471 (17)	0.39009 (16)	0.46840 (17)	0.0218 (4)	
C8	0.14351 (17)	0.42339 (16)	0.56916 (17)	0.0210 (4)	
C9	0.14867 (19)	0.52326 (16)	0.58418 (19)	0.0271 (5)	
C10	0.1866 (2)	0.56020 (17)	0.6785 (2)	0.0301 (5)	
C11	0.22151 (19)	0.49922 (17)	0.76248 (19)	0.0279 (5)	
C12	0.21817 (17)	0.40256 (16)	0.74993 (17)	0.0215 (4)	
C13	0.17848 (17)	0.36031 (15)	0.65197 (16)	0.0187 (4)	
C14	0.2753 (2)	0.3763 (2)	0.92607 (18)	0.0356 (6)	
C15	0.09237 (17)	0.03043 (17)	0.40283 (17)	0.0226 (4)	
C16	0.12100 (17)	−0.03338 (16)	0.48546 (17)	0.0206 (4)	
C17	0.11515 (19)	−0.13206 (16)	0.46470 (19)	0.0261 (5)	
C18	0.14356 (19)	−0.19674 (17)	0.5414 (2)	0.0293 (5)	
C19	0.17852 (19)	−0.16544 (17)	0.64144 (19)	0.0270 (5)	
C20	0.18411 (18)	−0.07012 (16)	0.66371 (17)	0.0227 (4)	
C21	0.15525 (17)	0.00007 (15)	0.58599 (16)	0.0193 (4)	
C22	0.2476 (2)	−0.09938 (19)	0.84100 (19)	0.0353 (6)	
C23	0.38345 (19)	0.14048 (19)	0.78310 (18)	0.0298 (5)	
C24	0.1234 (2)	0.1508 (2)	0.8408 (2)	0.0408 (6)	
H3	0.0505	0.0810	0.2100	0.033*	
H4	−0.0033	0.1877	0.0754	0.039*	
H5	−0.0272	0.3476	0.1043	0.038*	
H6	0.0162	0.4052	0.2687	0.033*	
H7	0.0844	0.4363	0.4164	0.026*	
H9	0.1254	0.5646	0.5277	0.033*	
H10	0.1896	0.6271	0.6879	0.036*	
H11	0.2476	0.5253	0.8284	0.034*	
H14A	0.2969	0.3247	0.9754	0.043*	
H14B	0.2125	0.4084	0.9323	0.043*	
H14C	0.3339	0.4219	0.9385	0.043*	
H15	0.0687	0.0036	0.3372	0.027*	
H17	0.0912	−0.1534	0.3968	0.031*	
H18	0.1396	−0.2628	0.5268	0.035*	
H19	0.1986	−0.2106	0.6946	0.032*	
H22A	0.2687	−0.0645	0.9049	0.042*	
H22B	0.3081	−0.1372	0.8375	0.042*	
H22C	0.1877	−0.1415	0.8368	0.042*	
H23A	0.3633	0.2061	0.7942	0.036*	
H23B	0.3177	0.1015	0.7605	0.036*	
H24A	0.1258	0.2182	0.8216	0.049*	
H24B	0.1414	0.1111	0.7908	0.049*	

### Atomic displacement parameters (Å^2^)

**Table:**

	*U*^11^	*U*^22^	*U*^33^	*U*^12^	*U*^13^	*U*^23^
Co1	0.02030 (15)	0.01570 (15)	0.01475 (14)	0.00061 (12)	0.00574 (11)	0.00093 (11)
Cl1	0.0392 (3)	0.0514 (4)	0.0337 (3)	0.0015 (3)	0.0199 (2)	0.0022 (3)
Cl2	0.0516 (4)	0.1169 (8)	0.0295 (3)	0.0179 (5)	0.0088 (3)	0.0173 (4)
Cl3	0.0503 (4)	0.0594 (5)	0.0615 (5)	0.0012 (4)	−0.0009 (3)	0.0198 (4)
Cl4	0.0392 (3)	0.0473 (4)	0.0512 (4)	−0.0109 (3)	0.0223 (3)	−0.0102 (3)
O1	0.0281 (8)	0.0165 (7)	0.0177 (7)	0.0011 (6)	0.0075 (6)	0.0001 (6)
O2	0.0258 (8)	0.0177 (7)	0.0178 (7)	−0.0001 (6)	0.0062 (6)	0.0002 (6)
O3	0.0367 (9)	0.0249 (8)	0.0172 (7)	0.0024 (7)	0.0066 (6)	−0.0028 (6)
O4	0.0449 (10)	0.0263 (9)	0.0203 (8)	0.0014 (7)	0.0089 (7)	0.0072 (6)
N1	0.0183 (8)	0.0200 (9)	0.0176 (8)	0.0018 (7)	0.0064 (7)	0.0030 (7)
N2	0.0185 (8)	0.0214 (9)	0.0160 (8)	0.0001 (7)	0.0059 (6)	0.0011 (7)
C1	0.0170 (10)	0.0269 (11)	0.0164 (10)	−0.0008 (8)	0.0050 (8)	0.0029 (8)
C2	0.0172 (10)	0.0277 (12)	0.0168 (10)	−0.0018 (8)	0.0051 (8)	0.0024 (8)
C3	0.0299 (12)	0.0314 (13)	0.0199 (11)	−0.0034 (10)	0.0061 (9)	−0.0020 (9)
C4	0.0300 (13)	0.0490 (16)	0.0174 (11)	−0.0070 (11)	0.0049 (9)	0.0015 (10)
C5	0.0289 (12)	0.0431 (15)	0.0197 (11)	−0.0016 (11)	0.0046 (9)	0.0106 (10)
C6	0.0264 (12)	0.0315 (12)	0.0236 (11)	0.0013 (10)	0.0077 (9)	0.0068 (10)
C7	0.0212 (10)	0.0225 (11)	0.0223 (11)	0.0021 (9)	0.0082 (8)	0.0062 (8)
C8	0.0193 (10)	0.0189 (11)	0.0269 (11)	0.0006 (8)	0.0104 (9)	0.0006 (9)
C9	0.0289 (12)	0.0197 (11)	0.0326 (13)	0.0016 (9)	0.0103 (10)	0.0029 (9)
C10	0.0347 (13)	0.0162 (11)	0.0401 (14)	−0.0005 (10)	0.0135 (11)	−0.0049 (10)
C11	0.0282 (12)	0.0244 (12)	0.0312 (12)	−0.0008 (10)	0.0099 (10)	−0.0094 (10)
C12	0.0205 (10)	0.0224 (11)	0.0230 (10)	0.0011 (9)	0.0090 (8)	−0.0016 (9)
C13	0.0168 (10)	0.0188 (11)	0.0220 (10)	0.0008 (8)	0.0084 (8)	−0.0006 (8)
C14	0.0459 (15)	0.0377 (14)	0.0208 (11)	0.0024 (12)	0.0078 (11)	−0.0074 (10)
C15	0.0211 (11)	0.0250 (11)	0.0224 (11)	−0.0022 (9)	0.0083 (8)	−0.0042 (9)
C16	0.0183 (10)	0.0208 (11)	0.0236 (10)	−0.0014 (8)	0.0084 (8)	−0.0010 (9)
C17	0.0270 (12)	0.0207 (11)	0.0321 (12)	−0.0029 (9)	0.0119 (10)	−0.0066 (9)
C18	0.0299 (13)	0.0179 (11)	0.0429 (14)	−0.0014 (10)	0.0162 (11)	−0.0013 (10)
C19	0.0283 (12)	0.0192 (11)	0.0361 (13)	0.0025 (9)	0.0141 (10)	0.0089 (9)
C20	0.0205 (11)	0.0223 (11)	0.0267 (11)	0.0007 (9)	0.0099 (9)	0.0044 (9)
C21	0.0167 (10)	0.0169 (10)	0.0252 (11)	0.0004 (8)	0.0084 (8)	0.0011 (8)
C22	0.0393 (14)	0.0388 (15)	0.0277 (12)	0.0043 (12)	0.0109 (11)	0.0156 (11)
C23	0.0251 (12)	0.0379 (14)	0.0265 (12)	−0.0005 (10)	0.0090 (9)	−0.0011 (10)
C24	0.0342 (14)	0.0486 (17)	0.0425 (15)	0.0010 (13)	0.0169 (12)	0.0072 (13)

### Geometric parameters (Å, °)

**Table:**

Co1—O1	1.8593 (15)	C12—C13	1.432 (3)
Co1—O2	1.8611 (14)	C15—C16	1.418 (3)
Co1—N1	1.8807 (17)	C16—C17	1.417 (3)
Co1—N2	1.8755 (18)	C16—C21	1.416 (3)
Cl1—C23	1.764 (3)	C17—C18	1.366 (3)
Cl2—C23	1.764 (2)	C18—C19	1.400 (3)
Cl3—C24	1.764 (2)	C19—C20	1.375 (3)
Cl4—C24	1.757 (3)	C20—C21	1.429 (3)
O1—C13	1.304 (2)	C3—H3	0.950
O2—C21	1.307 (2)	C4—H4	0.950
O3—C12	1.367 (2)	C5—H5	0.950
O3—C14	1.426 (2)	C6—H6	0.950
O4—C20	1.367 (2)	C7—H7	0.950
O4—C22	1.428 (3)	C9—H9	0.950
N1—C1	1.423 (2)	C10—H10	0.950
N1—C7	1.306 (2)	C11—H11	0.950
N2—C2	1.426 (2)	C14—H14A	0.980
N2—C15	1.307 (3)	C14—H14B	0.980
C1—C2	1.393 (3)	C14—H14C	0.980
C1—C6	1.395 (3)	C15—H15	0.950
C2—C3	1.393 (3)	C17—H17	0.950
C3—C4	1.386 (3)	C18—H18	0.950
C4—C5	1.386 (3)	C19—H19	0.950
C5—C6	1.386 (3)	C22—H22A	0.980
C7—C8	1.418 (3)	C22—H22B	0.980
C8—C9	1.421 (3)	C22—H22C	0.980
C8—C13	1.416 (3)	C23—H23A	0.990
C9—C10	1.358 (3)	C23—H23B	0.990
C10—C11	1.409 (3)	C24—H24A	0.990
C11—C12	1.372 (3)	C24—H24B	0.990
			
O1—Co1—O2	86.60 (6)	O2—C21—C20	118.52 (19)
O1—Co1—N1	94.10 (7)	C16—C21—C20	116.76 (19)
O1—Co1—N2	175.97 (8)	Cl1—C23—Cl2	110.01 (14)
O2—Co1—N1	177.70 (7)	Cl3—C24—Cl4	111.07 (18)
O2—Co1—N2	94.02 (7)	C2—C3—H3	120.2
N1—Co1—N2	85.43 (7)	C4—C3—H3	120.2
Co1—O1—C13	127.89 (13)	C3—C4—H4	119.9
Co1—O2—C21	127.82 (13)	C5—C4—H4	119.9
C12—O3—C14	115.96 (18)	C4—C5—H5	119.7
C20—O4—C22	117.44 (18)	C6—C5—H5	119.7
Co1—N1—C1	113.43 (14)	C1—C6—H6	120.5
Co1—N1—C7	126.48 (14)	C5—C6—H6	120.5
C1—N1—C7	119.96 (18)	N1—C7—H7	117.4
Co1—N2—C2	113.23 (14)	C8—C7—H7	117.4
Co1—N2—C15	126.50 (15)	C8—C9—H9	119.7
C2—N2—C15	120.19 (18)	C10—C9—H9	119.7
N1—C1—C2	113.64 (18)	C9—C10—H10	120.1
N1—C1—C6	125.9 (2)	C11—C10—H10	120.1
C2—C1—C6	120.4 (2)	C10—C11—H11	119.7
N2—C2—C1	114.06 (19)	C12—C11—H11	119.7
N2—C2—C3	126.2 (2)	O3—C14—H14A	109.5
C1—C2—C3	119.7 (2)	O3—C14—H14B	109.5
C2—C3—C4	119.7 (2)	O3—C14—H14C	109.5
C3—C4—C5	120.3 (2)	H14A—C14—H14B	109.5
C4—C5—C6	120.7 (2)	H14A—C14—H14C	109.5
C1—C6—C5	119.1 (2)	H14B—C14—H14C	109.5
N1—C7—C8	125.2 (2)	N2—C15—H15	117.2
C7—C8—C9	117.4 (2)	C16—C15—H15	117.2
C7—C8—C13	121.8 (2)	C16—C17—H17	119.7
C9—C8—C13	120.8 (2)	C18—C17—H17	119.7
C8—C9—C10	120.6 (2)	C17—C18—H18	120.1
C9—C10—C11	119.9 (2)	C19—C18—H18	120.1
C10—C11—C12	120.6 (2)	C18—C19—H19	119.6
O3—C12—C11	124.5 (2)	C20—C19—H19	119.6
O3—C12—C13	113.98 (18)	O4—C22—H22A	109.5
C11—C12—C13	121.5 (2)	O4—C22—H22B	109.5
O1—C13—C8	124.51 (19)	O4—C22—H22C	109.5
O1—C13—C12	118.94 (18)	H22A—C22—H22B	109.5
C8—C13—C12	116.54 (19)	H22A—C22—H22C	109.5
N2—C15—C16	125.6 (2)	H22B—C22—H22C	109.5
C15—C16—C17	118.1 (2)	Cl1—C23—H23A	109.7
C15—C16—C21	121.2 (2)	Cl1—C23—H23B	109.7
C17—C16—C21	120.7 (2)	Cl2—C23—H23A	109.7
C16—C17—C18	120.6 (2)	Cl2—C23—H23B	109.7
C17—C18—C19	119.8 (2)	H23A—C23—H23B	108.2
C18—C19—C20	120.8 (2)	Cl3—C24—H24A	109.4
O4—C20—C19	124.5 (2)	Cl3—C24—H24B	109.4
O4—C20—C21	114.12 (19)	Cl4—C24—H24A	109.4
C19—C20—C21	121.4 (2)	Cl4—C24—H24B	109.4
O2—C21—C16	124.72 (19)	H24A—C24—H24B	108.0
			
O1—Co1—O2—C21	−176.86 (19)	N2—C2—C3—C4	177.9 (2)
O2—Co1—O1—C13	−178.94 (19)	C1—C2—C3—C4	−3.4 (3)
O1—Co1—N1—C1	176.77 (15)	C2—C3—C4—C5	−0.9 (3)
O1—Co1—N1—C7	1.0 (2)	C3—C4—C5—C6	3.2 (4)
N1—Co1—O1—C13	−1.1 (2)	C4—C5—C6—C1	−1.1 (3)
O2—Co1—N2—C2	179.84 (15)	N1—C7—C8—C9	−179.9 (2)
O2—Co1—N2—C15	3.2 (2)	N1—C7—C8—C13	1.7 (3)
N2—Co1—O2—C21	−0.85 (19)	C7—C8—C9—C10	−178.9 (2)
N1—Co1—N2—C2	2.08 (15)	C7—C8—C13—O1	−1.9 (3)
N1—Co1—N2—C15	−174.6 (2)	C7—C8—C13—C12	178.6 (2)
N2—Co1—N1—C1	0.78 (16)	C9—C8—C13—O1	179.8 (2)
N2—Co1—N1—C7	−175.0 (2)	C9—C8—C13—C12	0.3 (3)
Co1—O1—C13—C8	1.8 (3)	C13—C8—C9—C10	−0.5 (3)
Co1—O1—C13—C12	−178.74 (16)	C8—C9—C10—C11	0.2 (3)
Co1—O2—C21—C16	−1.1 (3)	C9—C10—C11—C12	0.4 (4)
Co1—O2—C21—C20	179.16 (16)	C10—C11—C12—O3	−179.6 (2)
C14—O3—C12—C11	7.9 (3)	C10—C11—C12—C13	−0.6 (3)
C14—O3—C12—C13	−171.2 (2)	O3—C12—C13—O1	−0.1 (2)
C22—O4—C20—C19	1.1 (3)	O3—C12—C13—C8	179.4 (2)
C22—O4—C20—C21	−179.3 (2)	C11—C12—C13—O1	−179.3 (2)
Co1—N1—C1—C2	−3.6 (2)	C11—C12—C13—C8	0.3 (3)
Co1—N1—C1—C6	177.4 (2)	N2—C15—C16—C17	−178.4 (2)
Co1—N1—C7—C8	−1.4 (3)	N2—C15—C16—C21	1.2 (3)
C1—N1—C7—C8	−177.0 (2)	C15—C16—C17—C18	178.8 (2)
C7—N1—C1—C2	172.6 (2)	C15—C16—C21—O2	1.3 (3)
C7—N1—C1—C6	−6.5 (3)	C15—C16—C21—C20	−178.9 (2)
Co1—N2—C2—C1	−4.6 (2)	C17—C16—C21—O2	−179.1 (2)
Co1—N2—C2—C3	174.2 (2)	C17—C16—C21—C20	0.7 (3)
Co1—N2—C15—C16	−3.7 (3)	C21—C16—C17—C18	−0.7 (3)
C2—N2—C15—C16	179.8 (2)	C16—C17—C18—C19	0.2 (3)
C15—N2—C2—C1	172.3 (2)	C17—C18—C19—C20	0.4 (4)
C15—N2—C2—C3	−8.9 (3)	C18—C19—C20—O4	179.3 (2)
N1—C1—C2—N2	5.3 (2)	C18—C19—C20—C21	−0.4 (3)
N1—C1—C2—C3	−173.6 (2)	O4—C20—C21—O2	−0.0 (2)
N1—C1—C6—C5	175.8 (2)	O4—C20—C21—C16	−179.8 (2)
C2—C1—C6—C5	−3.2 (3)	C19—C20—C21—O2	179.7 (2)
C6—C1—C2—N2	−175.6 (2)	C19—C20—C21—C16	−0.1 (2)
C6—C1—C2—C3	5.5 (3)		

### Hydrogen-bond geometry (Å, °)

**Table:**

*D*—H···*A*	*D*—H	H···*A*	*D*···*A*	*D*—H···*A*
C23—H23A···O3	0.99	2.54	3.472 (4)	158
C23—H23B···O4	0.99	2.33	3.252 (4)	154
C23—H23B···O2	0.99	2.41	3.202 (3)	137
C24—H24A···O3	0.99	2.35	3.173 (4)	140
C24—H24B···O4	0.99	2.38	3.250 (4)	147

## References

[bb1] Altomare, A., Burla, M. C., Camalli, M., Cascarano, G. L., Giacovazzo, C., Guagliardi, A., Moliterni, A. G. G., Polidori, G. & Spagna, R. (1999). *J. Appl. Cryst.* **32**, 115–119.

[bb2] Bella, S. D., Fragala, I., Ledoux, I. & Marks, T. J. (1995). *J. Am. Chem. Soc.* **117**, 9481–9485.

[bb3] Cotton, F. A., Wilkinson, G., Murillo, C. A. & Bochmann, M. (1999). *Advanced Inorganic Chemistry*, 6th ed., ch. 17F. New York: Wiley-Interscience.

[bb4] Jacobson, R. (1998). *REQAB* Iowa State University, USA.

[bb5] Johnson, C. K. (1976). *ORTEPII* Report ORNL-5138. Oak Ridge National Laboratory, Tennessee, USA.

[bb6] Liu, J.-M., Peng, X.-G., Liu, J.-H., Zheng, S.-Z., Sun, W. & Xia, C.-G. (2007). *Tetrahedron Lett.* **48**, 929–932.

[bb7] Pahor, N. B., Calligaris, M., Delise, P., Dodic, G., Nardin, G. & Randaccio, L. (1976). *J. Chem. Soc. Dalton Trans.* pp. 2478–2483.

[bb8] Rigaku/MSC (2007). *CrystalStructure* and *CrystalClear* Rigaku/MSC, The Woodlands, Texas, USA.

[bb9] Sharghi, H. & Ali Nasseri, M. (2003). *Bull. Chem. Soc. Jpn*, **76**, 137–142.

[bb10] Sheldrick, G. M. (2008). *Acta Cryst.* A**64**, 112–122. 10.1107/S0108767307043930 18156677

